# Health-risk assessment of Portuguese man-of-war (*Physalia physalis*) envenomations on urban beaches in São Luís city, in the state of Maranhão, Brazil

**DOI:** 10.1590/0037-8682-0216-2020

**Published:** 2020-09-25

**Authors:** Mayana Mendes e Silva Cavalcante, Zulimar Márita Ribeiro Rodrigues, Rachel Ann Hauser-Davis, Salvatore Siciliano, Vidal Haddad, Jorge Luiz Silva Nunes

**Affiliations:** 1 Universidade Federal do Maranhão, Programa de Pós-Graduação em Saúde e Ambiente, São Luís, MA, Brasil.; 2 Universidade Federal do Maranhão, Núcleo de Estudos e Pesquisas Ambientais, São Luís, MA, Brasil.; 3 Instituto Oswaldo Cruz/Fiocruz, Laboratório de Avaliação e Promoção da Saúde Ambiental, Rio de Janeiro, RJ, Brasil.; 4 Instituto Oswaldo Cruz/Fiocruz, Laboratório de Biodiversidade, Rio de Janeiro, RJ, Brasil.; 5 Universidade Estadual Paulista Júlio de Mesquita Filho, Departamento de Dermatologia e Radioterapia, Botucatu, SP, Brasil.; 6 Universidade Federal do Maranhão, Departamento de Oceanografia e Limnologia, São Luís, MA, Brasil.

**Keywords:** Physalia physalis, Geographic mapping, Venomous animals, Injury prevention, Public health

## Abstract

**INTRODUCTION:**

The Portuguese man-of-war (*Physalia physalis*) is a cosmopolitan species, with a widespread distribution and responsible for a great number of injuries caused by cnidarians worldwide, including Brazil. Geoprocessing technology, however, has never been used to assess the spatial distribution of these animals on beaches. The aim of this study was to carry out a health risk assessment of Portuguese man-of-war (*P. physalis*) envenomations on the São Marcos and Calhau beaches in São Luís city, in the state of Maranhão, Brazil.

**METHODS:**

This is a descriptive and quantitative study concerning primary data on the occurrence of the Portuguese man-of-war (*P. physalis*) and human envenomations in the studied places, conducted over a two-year period in São Luís, Maranhão, northeastern Brazil.

**RESULTS:**

Envenomations mainly occurred on beaches presenting high density of *P. physalis* during the dry period. Vinegar has been incorporated as a first aid, according to recommendations set by the Brazilian Ministry of Health.

**CONCLUSIONS:**

In order to improve prevention and control actions of human envenomation, risk areas for this type of envenomation should be clearly indicated as alert areas. Inclusion of the geographical location of the envenomation in the Notification/Investigation SINAN Form was suggested for allowing the continuity of studies involving this public health issue.

## INTRODUCTION

The Portuguese man-of-war (*Physalia physalis*) is a conspicuous colonial cnidarian with a bluish-pink coloration. It presents a gas vesicle called a pneumatophore with a triangular shape and folds in the upper portion^1^. Its multiple tentacles are capable of firing thousands of intracellular organelles (cnida) filled with venom and used for predation or defense. This species is responsible for a high number of envenomations, which may lead to severe injuries among humans and even death^2^ ^-^ ^4^.

The pneumatophore enables these organisms to move either by drifting with currents or sailing on wind using the crest of its floater as a sail. The geographical distribution is cosmopolitan and widespread, throughout the Indian Ocean and the South Atlantic^5^ ^,^ ^6^.

*P. physalis* causes several socioeconomic problems in countries such as New Zealand, Australia, Portugal, Mexico, France, Spain, Chile^7^, and Brazil, due to human envenomations and consequent impacts on the local tourist economy^8^ ^-^ ^10^.

The Portuguese man-of-war is recognized as a cause of envenomation in bathers, mainly in northern and northeastern Brazil^4^ ^,^ ^7^ ^,^ ^11^ ^-^ ^14^. Colonies have been registered along the entire Brazilian coast^15^ ^,^ ^16^, and envenomation outbreak records have been described in the states of Rio Grande do Sul, Paraná, São Paulo, Rio de Janeiro, Pernambuco and Maranhão^7^ ^,^ ^8^ ^,^ ^11^.

The clinical manifestations of cnidarian injuries are typical, with the appearance of linear plaques comprising edema and erythema and intense pain immediately after contact with the animals on beaches. Systemic phenomena are rare, although dyspnea, malaise, nausea, vomiting, headache, chills, drowsiness, arterial hypotension, and cardiac arrhythmias have been reported^2^ ^,^ ^5^ ^,^ ^11^ ^,^ ^14^ ^-^ ^19^. In one case, an anomalous reaction manifested by purpuric papules was also observed after the initial phase of envenoming^13^.

 In Brazil, envenomations caused by venomous animals are included in the Compulsory Notification List (*Lista de Notificação Compulsória* - LNC) as set by Ordinance 204 from February 17^th^, 2016 by the Brazilian Health Ministry, and must be notified and recorded in the Injury Notification Information System (*Sistema de Informação de Agravos de Notificação* - SINAN)^20^. However, Portuguese man-of-war envenomation cases registered in the SINAN indicate important sub-notifications compared to regional and local surveys. Due to a gap in the epidemiology of accidents caused by aquatic animals in Brazil, the limited information available in the literature is the result of cross-sectional studies, active case searches or research in medical files, presenting several restrictions inherent to the collection of secondary data^21^. This, therefore, makes it difficult to accurately determine the extent of the problem in the country and plan preventive actions.

Portuguese man-of-war envenomations are common on the beaches of São Luís, in the state of Maranhão, Brazil, particularly during the second half of the year, when these organisms are frequently found. However, few actions have been carried out to alert the population about preventive measures and prompt care in case of accidental contact with these animals^19^ ^,^ ^22^. Since injured people search for the lifeguard department as the first location for first-aid measures, the Marine Fire Brigade (*Batalhão de Bombeiros Marinhos* - BBMar) health team and fire brigade need to be notified of local envenomations. These data, however, are not present in the SINAN database.

In this regard, geoprocessing technology has never been used to assess the distribution of these animals and human envenomations in São Luís, and may prove a valuable tool in this context. Thus, the aim of this study was to assess the emerging health risk due to Portuguese man-of-war (*P. physalis*) envenomation on the São Marcos and Calhau beaches in São Luís, Maranhão, Brazil during the years 2015 and 2016.

## METHODS

**FIGURE 1:**
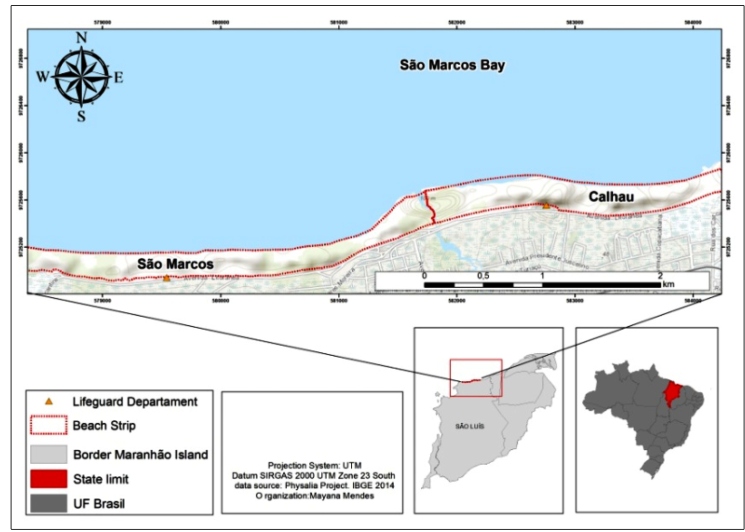
Map of the study area indicating the São Marcos and Calhau beaches, São Luís, Maranhão, Brazil.

This was a descriptive and quantitative study concerning primary data on the occurrence of the Portuguese man-of-war (*P. physalis*) and cases of human envenomation. About 7.0 km were monitored, considering the extension of the São Marcos (2°29’17.70” S, 44°17’04.53” W) and Calhau (2°28’58.65” S, 44°15’15.61” W) urban beaches, in São Luís, Maranhão, Brazil, during 2015 and 2016 (Figure 1).

The data were obtained by means of an intensive search procedure parallel to the tide line for counting *P. physalis* stranded in the sand and marking their geographical coordinates using a portable global positioning system device. Sampling was conducted every fortnight, at weekends and at the flood tide, as these animals are more frequently found in this type of tide. The data were geoprocessed using the QGIS version 16.1 software and the kernel density estimator was used to identify and analyze case concentrations and to statistically validate the risk of envenomation events through geolocality proximity calculations.

Information concerning envenomations caused by Portuguese man-of-war was obtained through the application of a specific questionnaire. These data were collected in lifeguard department posts on the two evaluated beaches, from August 2015 to December 2016, on weekends and holidays, when the number of beachgoers was higher. Data on envenomation (place of occurrence, month, year, and first-aid measures) were obtained.

### Ethical considerations

The study protocol was approved by the Ethics Committee of the Federal University of Maranhão (no. 1.625.949), and met the ethical principles for conducting research involving human beings, according to the Brazilian National Health Council (*Conselho Nacional de Saúde* - CNS) resolution no. 466/2012.

## RESULTS

**FIGURE 2:**
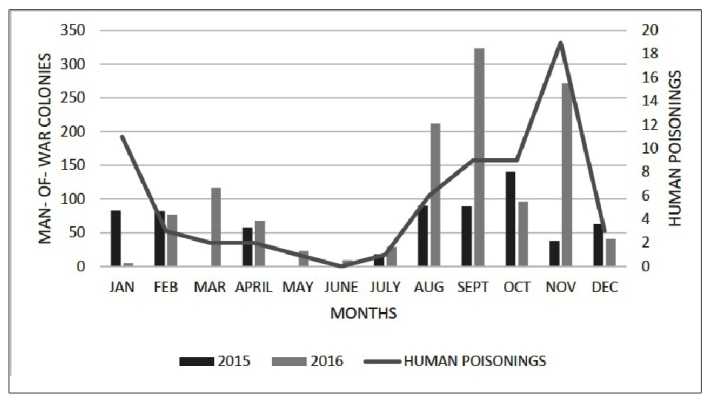
Temporal distribution of Portuguese man-of-war (*Physalia physalis*) specimens and human envenomation cases on Calhau and São Marcos beaches, São Luís, Maranhão, in 2015 and 2016.

From January 2015 to December 2016, a total of 1,929 Portuguese man-of-war specimens were found on the two evaluated beaches, 663 in 2015 and 1,273 in 2016. Portuguese man-of-war were most frequent in August (90), September (89), and October (140) 2015, and in August (212), September (324), and November (272) 2016 (Figure 2).

 A total of 66 human envenomations were identified from August 2015 to December 2016: 27 in Calhau and 39 in São Marcos. The months with the highest number of occurrences were January (11 cases), September (9 cases), and November (19 cases) (Figure 2).

**FIGURE 3:**
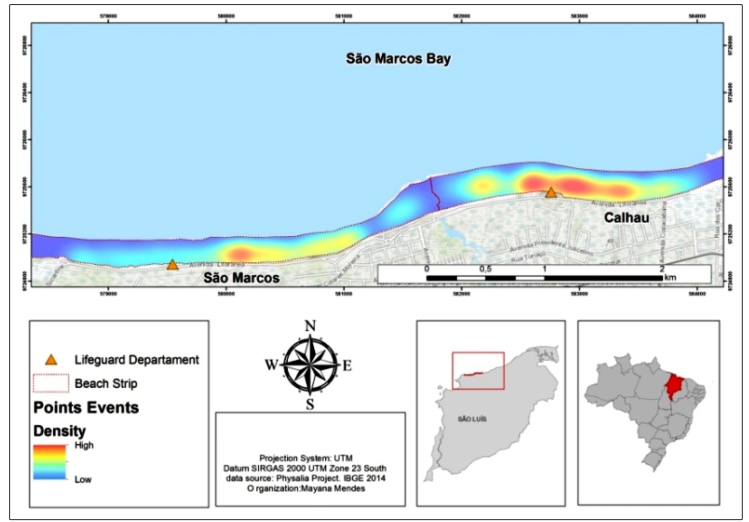
Distribution map of Portuguese man-of-war (*Physalia physalis*) specimens on Calhau and São Marcos beaches, São Luís, Maranhão, in 2015 and 2016.

Calhau beach presented a high density of Portuguese man-of-war specimens (1,002 organisms) located near the Calhau lifeguard station (between 2º28'53.980''S, 44º15'6.005"W and 2°28'55,200"S, 44°14'57.152"W), while São Marcos presented a density of 927 organisms, mostly concentrated on the stretch between 2º28'55,200''S, 44º14'57,152''W and 2º 28 '55,200"S, 44°14 '57.152 "W (Figure 3).

**FIGURE 4:**
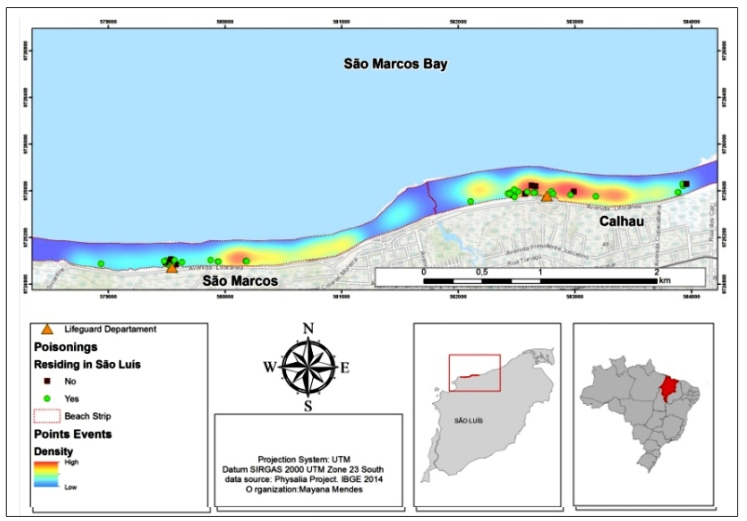
Map indicating the distribution of human Portuguese man-of-war (*Physalia physalis*) envenomation cases on Calhau and São Marcos beaches, São Luís, Maranhão, from August 2015 to December 2016.

The highest occurrence of Portuguese man-of-war specimens coincided with the highest occurrence of human envenomations on Calhau beach, as both are close to the lifeguard station. On the other hand, the area presenting the highest occurrence of Portuguese man-of-war colonies at São Marcos did not coincide with the area with the highest envenomation frequency, which was near the fire brigade. A total of 47 envenomations occurred in people living in São Luís and 19 in non-residents (Figure 4).

The main response measures adopted after Portuguese man-of-war envenomations at the lifeguard stations were as follows: topical vinegar (acetic acid) application at the site (50 cases); freshwater, silver sulphadiazine, and lidocaine (4 cases); vinegar and silver sulphadiazine (2 cases); freshwater and vinegar (2 cases); vinegar, silver sulphadiazine, and lidocaine application (1 case); freshwater, vinegar and sea water (1 case) and vinegar and sea water (1 case). Moreover, 5 cases did not receive first-aid measures. The bathers themselves carried out all the freshwater applications reported herein. 

## DISCUSSION

The high number of *P. physalis* organisms found during the two years of this assessment indicate a risk of human envenomations on the beaches in the study region. The largest Portuguese man-of-war agglomerations generally occur during the dry season (August to December), as reported previously by Haddad *et al.* ^11^in Brazil, Loten *et al.* ^23^ in Australia, Labadie *et al*.^9^ in France, Ferrer *et al.* ^24^ in Spain, and Araya *et al.* ^25^ in Chile.

Some studies point out that the environmental conditions that surround the Portuguese man-of-war agglomerations are not yet well known^15^, and the most common explanations for coastal agglomerations comprise associations between drought periods and transport phenomena (*e.g.* winds, ocean currents)^6^ ^,^ ^24^ ^,^ ^26^.

Portuguese man-of-war agglomerations have been reported in São Luís during periods of drought and strong winds (trade winds) present in the second half of the year, as reported by Ferreira-Bastos *et al.* ^19^ and Nunes and Mendonça^22^, which may explain most of the *P. physalis* agglomerations reported herein. In addition, Maranhão is located in the *P. physalis* distribution area range of the tropical zone^1^ ^,^ ^27^.

Furthermore, the high marine primary production in Maranhão promotes high marine trophic web productivity^28^ ^,^ ^29^, attracting several species to this region, may also be a cause for Portuguese man-of-war agglomerations, as *P. physalis* are predatory organisms and require surplus food resources to increase their frequency in certain locations^30^.

The higher density sites of Portuguese man-of-war organisms coincided with the sites where the greatest number of human envenomations occurred, while the months comprising the highest *P. physalis* sampling coincided with the dry season, when more envenomations were noted. On the other hand, the greatest number of envenomations was also observed in January, the rainy season, which might be connected with the school holidays, and the increased number of people on the beaches, as reported previously by Ferreira-Bastos *et al*.^19^. 

The highest frequency of injured people was noted at São Marcos beach, even with a lower Portuguese man-of-war density compared to the Calhau beach. Envenomation records indicate that most cases occurred near the lifeguard department, probably due to search for treatment at the nearest location to the envenomation site. This is important so that treatment measures can be carried out successfully. In the present study, the first-aid measures applied by the lifeguard department correspond to the Brazilian Ministry of Health recommendations. However, it was not always this way. In 2013, Ferreira-Bastos *et al*.^19^reported that the first-aid measures applied by the lifeguard department did not correspond to the Brazilian Ministry of Health recommendations.

After envenomation prevention actions developed by the “*Physalia* Project” popularly known in the region as the “Portuguese man-of-war Project”, a change in first-aid measures was applied at São Luís beaches. The main use of vinegar was incorporated into first-aid, as evidenced herein, in agreement with the Brazilian Ministry of Health recommendations. The use of silver sulfadiazine, lidocaine, and the application of freshwater, on the other hand, have no scientific basis and are contraindicated in cnidarian envenomation cases^4^.

It is interesting to note that most injured people were São Luís residents displaying little information about *P. physalis* or envenomation mechanisms^19^ ^,^ ^21^. Analyses of bathing conditions carried out by the Environmental State Department of Maranhão directly affect envenomation Portuguese man-of-war cases in urban São Luís beaches, as adequate signs on proper or improper bathing conditions lead to changes in the number of bathers in the area^19^.

 In addition, urban beaches in São Luís also suffer from intense anthropogenic impacts, with several domestic sewage discharge points along the study area, 36 in São Marcos and 15 in Calhau^31^ ^,^ ^32^, making these beaches less attractive to tourists, explaining most of the envenomations that occur among São Luís residents. This problem reflects not only health consequences but also tourism disturbances induced by the numerous beach closings, with huge economic impacts. Therefore, it does not only affect local authorities, but also local traders and beach goers.

According to Ferreira-Bastos *et al.* ^19^, injuries afflicted by Portuguese man-of-war specimens along the coast of São Luís have been reported as a real problem for bathers, although the authorities do not consider this a significant issue, despite compulsory notification being required and the fact that every year envenomation cases are reported during specific months. 

Despite this, the number of recorded envenomations in this study was low, perhaps due to media disclosure coverage on these events being common in São Luís^19^ ^,^ ^21^, probably instructing the population on the fact that prompt care is required. Other factors leading to a low number of reports may include the fact that local beach bar employees and owners have also been instructed on how to act in these situations, diluting lifeguard actions. Moreover, a lack of lifeguard awareness about the importance of notifications may lead to important sub-notification of human Portuguese man-of-war envenomations. 

By the end of 2016, the beaches of Calhau and São Marcos were marked with signs indicating adequate bathing areas, which may increase the number of Portuguese man-of-war envenomations. Thus, it is clear that understanding the spatial distribution of Portuguese man-of-war is paramount for the implementation of prevention measures and, in order to improve control actions, risk areas for this type of accident, namely the sections between 2º28'53.980''S, 44º15'6.005''W and 2º28'55.200''S, 44º14'57.152''W, on Calhau beach and 2º28'55.200''S, 44º14'57.152''W and 2º28'55.200''S, 44º14'57.152''W, on São Marcos beach, should be clearly indicated as alert areas, and the inclusion of the geographical location of each envenomation should be reported in the envenomation notification form, allowing for the continuity of studies involving this public health issue.

The study of Portuguese man-of-war envenomations has always been neglected by the agencies responsible for the knowledge, control and prevention of diseases involving these animals. Thus, we suggest this piece of evidence to the Surveillance Service with lifeguard teams for the purposes of updating and clinical training, as well as awareness about the importance of epidemiological data.

## References

[1] Bardi J, Marques AC (2007). Taxonomic redescription of the Portuguese man-of-war, Physalia
physalis (Cnidaria, Hydrozoa, Siphonophorae, Cystonectae) from
Brazil. Iheringia Ser. Zool.

[2] Fautin DG (2009). Structural diversity, systematic and evolution of
cnidae. Toxicon.

[3] Haddad V (2008). Animais aquáticos potencialmente perigosos do brasil: guia médico e
biológico.

[4] Haddad V, Virga R, Bechara A, Silveira FL, Morandini AC (2013). An outbreak of Portuguese man-of-war (Physalia physalis -
Linnaeus, 1758) envenoming in Southeastern Brazil. Rev Soc Bras Med Trop.

[5] Cegolon L, Heymann WC, Lange JH, Mastrangelo G (2013). Jellyfish stings and their management: a review. Mar Drugs.

[6] Mapstone GM (2014). Global Diversity and Review of Siphonophorae (Cnidaria:
Hydrozoa). PLoS One.

[7] Neves RF, Amaral FD, Steiner AQ (2007). Levantamento de registros dos acidentes com cnidários em algumas
praias do litoral de Pernambuco (Brasil). Cien Saude Colet.

[8] Bochner R, Struchiner CJ (2002). Acidentes por animais peçonhentos e sistemas nacionais de
informação. Cad Saude Publica.

[9] Labadie M, Aldabe B, Ong N, Joncquiert-Latarjet A, Groult V, Poulard A (2012). Portuguese man-of-war (Physalia physalis) envenomation on the
Aquitaine Coast of France: An emerging health risk. Clin Toxicol.

[10] General Fisheries Commission for the Mediterranean (GFCM) (2013). Food and Agriculture Organization of the United Nations Series
92. Review of jellyfish blooms in the mediterranean and black sea.

[11] Haddad V (2003). Aquatic animals of medical importance. Rev Soc Bras Med Trop.

[12] Ramírez MM, Zálvez MEV, Jara IM, Orden JM (2010). Picadura por Carabela Portuguesa, una “medusa” algo
especial. Rev Clin Med Fam.

[13] Risk YJ, Cardoso JLC, Haddad V (2012). Envenoming caused by a Portuguese man-of-war (Physalia physalis)
manifesting as purpuric papules. An Bras Dermatol.

[14] Moleiro S, Pereira A, Lopes MJP (2013). Dermatose Marítima por Contato com uma
Caravela-Portuguesa. Acta Med Port.

[15] Purcell JE, Uye SI, Lo WT (2007). Antropogenic causes of jellyfish blooms and their direct
consequences for humans: A review. Mar Ecol Prog Ser.

[16] Condon RH, Duarte CM, Pitt KA, Robinson KL, Lucas CH, Sutherland KR (2013). Recurrent jellyfish blooms are a consequence of global
oscillations. Proc Natl Acad Sci U S A.

[17] Haddad V, Silveira FL, Cardoso JLC, Morandini AC (2002). A report of 49 cases of cnidarian envenoming from southeastern
Brazilian coastal waters. Toxicon.

[18] Cardoso JLC, França FOS, Wen FH, Malaque CMS, Haddad V. (2003). Venomous animals in Brazil: biology, clinics and therapeutic of the
accidents.

[19] Ferreira-Bastos DMR, Haddad V, Nunes JLS (2017). Human envenomations caused by Portuguese man-of-war (Physalia
physalis) in urban beaches of São Luís City, Maranhão State, Northeast Coast
of Brazil. Rev Soc Bras Med Trop.

[20] Ministério da Saúde (MS) (2016). Portaria nº 204, de 17 de fevereiro de 2016. Define a Lista Nacional de
Notificação Compulsória de doenças, agravos e eventos de saúde pública nos
serviços de saúde públicos e privados em todo o território nacional, nos
termos do anexo, e dá outras providências.

[21] Reckziegel GC, Dourado FS, Neto DG, Haddad V (2015). Injuries caused by aquatic animals in Brazil: an analysis of the
data present in the information system for notifiable
diseases. Rev Soc Bras Med Trop.

[22] JlS Nunes, Mendonça MA (2013). Biodiversidade marinha da Ilha do Maranhão.

[23] Loten C, Stokes B, Worsley D, Seymour JE, Jiang S, Isbister GK (2006). A randomized controlled trial of hot water (45 ° C) immersion
versus ice packs for pain relief in bluebottle stings. Med J Aust.

[24] Ferrer L, Zaldua-Mendizabal AC, Franco J, Mader J, Cotano U, Uriarte A (2013). Protocolo operacional para el avistamento y seguimento del
cnidário Physalia physalis (carabela portuguesa) em el sureste del golfo de
Bizkaia. RIM-Revista Investig Mar.

[25] Araya JF, Aliaga JA, Araya ME (2016). On the distribution of Physalia physalis (Hydrozoa: Physaliidae)
in Chile. Mar Biodivers.

[26] Pontin DR, Schliebs S, Worner SP, Watts MJ (2011). Determining factors that inﬂuence the dispersal of a pelagic
species: A comparison between artiﬁcial neural networks and evolutionary
algorithms. Ecol Modell.

[27] Purcell JE (1984). Predation on fish larvae by Physalia physalis, the Portuguese man
of War. Mar Ecol Prog Ser.

[28] Azevedo ACG, Feitosa FAN, Koening ML (2008). Distribuição espacial e temporal da biomassa fito-planctônica e
variáveis ambientais no Golfão Maranhense, Brasil. São Luís, MA,
Brasil. Acta Bot Brasilica.

[29] Carvalho RCQ, Cutrim MVJ, Eschrique SA, Cutrim ACGA, Moreira EG, Silveira PCA (2016). Microphytoplankton composition, chlorophyll-a concentration and
environmental variables of the Maranhão Continental Shelf, Northern
Brazil. Lat Am J Aquat Res.

[30] Purcell JE (2005). Climate effects on formation of jellyfish and ctenophore blooms.
A review. J Mar Biol Assoc U K.

[31] Trindade WN, Pereira LCC, Guimarães DO, Silva IR, Costa RM (2011). The effects of sewage discharge on the water quality of the
beaches os São Luís (Maranhão, Brazil). J Coast Res.

[32] Silva IR, Pereira LCC, Trindade WN, Magalhães A, Costa RM (2013). Natural and anthropogenic processes on the recreational
activities in urban Amazon beaches. Ocean Coast Manag.

